# SSLVNet: an explainable multi-modal hybrid framework integrating self-supervised learning and vision transformers for multi-label classification of retinal diseases

**DOI:** 10.3389/frai.2026.1937520

**Published:** 2026-09-21

**Authors:** M. Rithiga, R. K. Nadesh

**Affiliations:** School of Computer Science Engineering and Information Systems, Vellore Institute of Technology, Vellore, Tamil Nadu, India

**Keywords:** classification, convolutional neural networks, explainable AI, lesion attention, retinal diseases, self-supervised learning, vision transformer

## Abstract

**Introduction:**

Retinal Diseases are the main reason for vision loss and blindness worldwide. Initial identification of retinal abnormalities is essential for avoiding severe retinal-related complications and improving patient results. Traditional retinal disease diagnosis mainly depends on manual checks of retinal fundus images, which are slow and highly dependent on medical expertise. Deep learning methods are used in retinal disease classification. Existing methods are primarily based on labelled image data and often do not fully utilize complementary clinical information. The proposed hybrid multi-model framework classifies retinal diseases from the available retinal fundus images.

**Methods:**

SSLVNet combines a Self-Supervised Learning encoder, Lesion Attention, CNN, ViT, and a Simplified lesion Graph. Also, Grad- CAM provides visual elucidations of the model predictions. ODIR-5K dataset, which includes multiple ocular disease categories, is used.

**Results:**

The results are promising, with an Exact Accuracy of 84.44%, a Hamming accuracy of 96.92%, and a Macro F1-Score of 90.36%. An ablation study was done to understand the contribution of individual components in the proposed framework.

**Discussion:**

These results indicate that the proposed work can effectively support reliable and interpretable retinal disease classification in real-world healthcare applications.

## 1 Introduction

Retinal Diseases like myopia, diabetic retinopathy, Cataract, hypertension, glaucoma, age-related macular degeneration, and abnormalities are complications of vision loss and can lead to wide-reaching blindness if they are not identified and treated at an early stage (Herrera-Chavez et al., 2024). Since many of these diseases develop slowly without noticeable symptoms, early screening becomes very important to avoid permanent vision loss. Transfer Learning and Deep Learning models have also demonstrated enhanced accuracy and robustness when applied across various retinal datasets (ul Hassan et al., 2024). Ophthalmologists use retinal fundus images for visualization of eye structures like blood vessels, the optic disc, and the macula (Shamsan et al., 2023). Retinal images are still analyzed manually by specialists. But this method takes too much time and may vary depending on the experience of the observer (Yu and Dong, 2025). Due to these limitations and growing demand, there is a need for automated systems to assist ophthalmologists in retinal disease classification, thus improving the screening efficiency (Zhang et al., 2021). In recent years, artificial intelligence techniques have been used in the analysis of medical images, especially for ophthalmic diagnosis (Shafiq et al., 2024). These methods are used to detect complex learning patterns from retinal images and have achieved good performance in identifying different eye diseases (Hu et al., 2025). Moreover, multi-label classification techniques are becoming more widely adopted because a single retinal image can display several diseases simultaneously (Aranha et al., 2023). AI-based approaches have also been explored for precision diagnosis and treatment planning in retinal diseases (Wang et al., 2024). These works show that deep learning can effectively extract important features from retinal images and support early diagnosis. Further improvements were made by introducing more advanced architectures for ocular image classification. A dual-stream feature transfer context was proposed for multi-model ocular image classification, which helped improve feature learning from different image sources (Annamalai and Palani, 2025). Many existing methods have some limitations. They use only retinal images and exclude clinical information, which can improve diagnosis. Most developed models focus only on a few diseases and cannot properly understand the relationships between different retinal problems. Another issue is that many systems are not easy to explain, so doctors may not fully trust them. Considering the gap mentioned, a hybrid multi-model framework is proposed that integrates SSL, CNN, ViT, lesion attention, and a Lesion Graph Network to extract more informative features from retinal images and to enhance disease classification. The model also employs Grad-CAM, which can highlight which region of the image is crucial for the prediction and make it more understandable. The proposed method is tested using the ODIR-5k dataset. The results show better performance and better interpretability compared to existing methods. Key contributions are,

- Multi-modal system for all retinal disease classification.
- Integrating Self-supervised Learning (SSL) for robust feature pattern Learning and a CNN-Vision Transformer hybrid architecture for local and global feature extraction.
- Lesion Graph Network (LGN) for modeling relationships among retinal abnormalities.
- Grad-CAM for Visualization.

## 2 Related work

In early research works, deep learning models were widely used for retinal structure analysis. For example, a retinal blood vessel segmentation framework was proposed to support a better understanding of retinal anatomy and disease classification (Al-Fahdawi et al., 2024). Similarly, an automated retinal Screening system was developed to identify different retinal abnormalities from fundus images (Wang, 2025). The researchers have focused on improving the practical usability of these systems. Low-cost and efficient AI- based approaches have been developed to support early detection or classification in resource-limited healthcare platforms (Vohra et al., 2025). In another study, a combined model with SVM and EfficientNet was introduced (Naveen et al., 2025). An innovative hybrid framework was proposed for DR detection, which improved feature extraction and also classified retinal diseases utilizing retinal vascular structures to enhance classification performance (Sushith et al., 2025). CAD-EYE, a multi-eye disease classification method, was implemented using feature fusion techniques and achieved good performance (Khalid et al., 2024). An explainable AI-based framework was introduced for detecting age-related macular degeneration, which helped in understanding model decisions and improving clinical trust (Wang et al., 2023). In addition, retinal lesion segmentation has also been widely studied. A deep learning-based model was proposed for accurate retinal lesion segmentation, which helped in identifying the disease regions more precisely (Kumar et al., 2023). Another study combined CycleGAN with CNN models to improve retinal pathology localization, which helped in better identifying disease regions in images (Zhang et al., 2022). Conv-ViT, a model that pairs two CNNs (Inception-V3, ResNet-50) with a Vision Transformer to analyze retinal OCT scans, catches fine texture details while the transformer picks up on broader shape and structure (Dutta et al., 2023). Additionally, a study was proposed for early-stage diagnosis to help in understanding disease progression (Sattigeri et al., 2022). In addition, automated retinal disease detection using Fundus images, focusing on reducing computation time while maintaining accuracy, was explored (Rajesh et al., 2025). Several researchers have also focused on improving disease classification using hybrid and advanced deep learning methods for AMD (Osa-Sanchez et al., 2025). Federated learning approaches have also been explored for healthcare applications to improve data privacy while training deep learning models (Bashir et al., 2023). It combines local and contextual features, which helps the model identify retinal abnormalities more effectively and improves retinal disease detection (Khan et al., 2025). Finally, CNN-based frameworks have been developed for initially improving eye disease identification, showing the usefulness of deep learning in retinal eye disease classification using Explainable AI (Ahnaf Alavee et al., 2024). Transfer learning with different CNN models, including various methods like VGG16, ResNet, InceptionV3, and MobileNet (Gour and Khanna, 2021). Several CNN models use bilateral feature fusion to improve feature representation (Li et al., 2021). It combines bilateral feature enhancement with multi-scale feature extraction for ocular disease classification (Ou et al., 2022). The multi-module attention-based framework helps the model focus on important regions, leading to better gastrointestinal disease identification (Khan et al., 2024). The closest contributions of the existing literature are summarized and given in Table 1.

**Table 1:** Comparison of existing methods with the proposed SSLVNet.

Author	Dataset	Labels	Method	Result
Gour and Khanna (2021)	ODIR	8 ocular disease classes	Transfer learning-based CNN	85.57% F1-score
Li et al. (2021)	OIA-ODIR	8 ocular disease classes	CNN-based bilateral feature fusion	87.93% F1-score
Ou et al. (2022)	OIA-ODIR	8 ocular disease classes	BFENet: two-stream interaction CNN	89.20% F1-score
Al-Fahdawi et al. (2024)	OIA-ODIR	8 ocular disease classes	Fundus-DeepNet: HRNet + Attention + SENet + DRBM	89.13% F1-score
Proposed SSLVNet	ODIR-5K	8 ocular disease classes	Self-Supervised Learning + Feature Fusion + Hierarchical Transformer + Lesion Graph Attention + Uncertainty Estimation	90.36% Macro F1-score

## 3 Proposed methodology

The proposed architecture is a Hybrid Multi-Model, designed and developed for automating retinal disease classification. The input is retinal fundus images. It processes and converts the data in the Self-Supervised Learning module. The data is processed from Unlabeled Retinal Images, and it is converted into Pretext tasks. Then, the CNN Dual function is used in feature extraction because CNNs are used in EfficientNet-B4, and another function are used in the Transformer backbone, which is the vision transformer (ViT-B/16). It includes local and global features like structure. The data is moved to feature fusion are include in CNN Features, ViT features. In the embedding, images will be converted to the embedding space for each patch, and a fully connected layer will be used to unify the feature vector for each patch. The Hierarchical transformer is also used in the proposed model because it can include the multi-head and self-attention layers used in the input paths. The Lesion Graph Network is a process of detecting the different nodes included in the graph. It detects the lesions of the graph attention network, and it can identify the inter-lesion spatial. Pseudo-lesion regions are obtained from the spatial attention map generated by the Lesion Attention module. The attention map is divided into a 7 × 7 grid, and the eight grid regions with the highest attention scores are selected as pseudo-lesion nodes. Then, classifying the retinal diseases can be done by using a fully connected layer and a sigmoid activation function to predict probabilities for each retinal disease. The final predicted classes are classified based on the confidence scores for the proposed model. A Grad-CAM heatmap image is used to denote the high and low importance of the image and is given in Figure 1.

**Figure 1:**
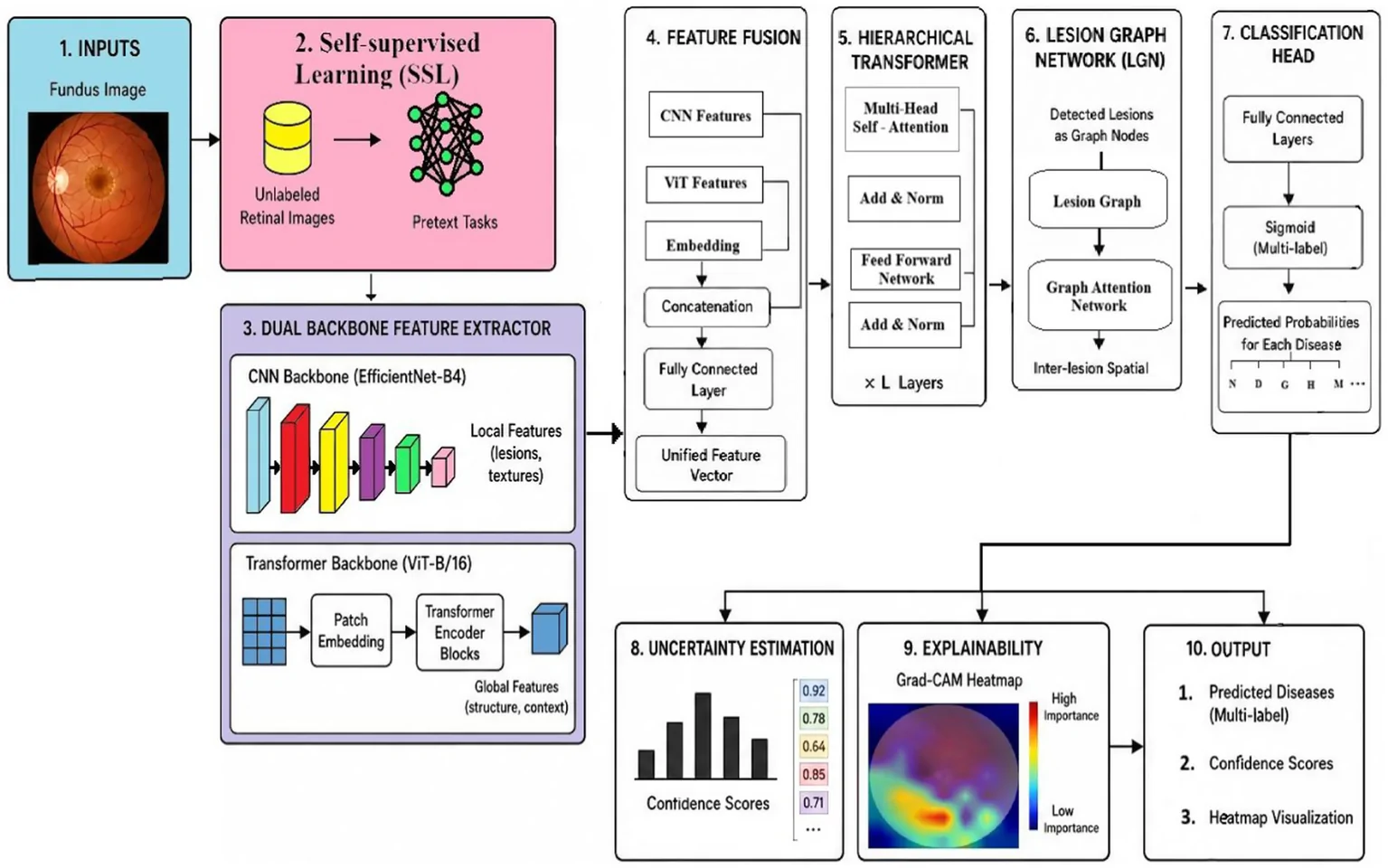
Hybrid multi-model architecture for retinal disease classification.

### 3.1 Input acquisition module

The first step of the proposed framework is to gather the retinal information from fundus modalities, such as retinal fundus images, which contain important information for retinal diseases. As shown in Equation 1, the input retinal image is represented as,


$$\text{SSLVNe}t_{\mathrm{inp}(x)}=\{\text{in}p_{f}\}$$ (1)

### 3.2 Self-supervised learning (SSL) module

Figure 2, The second step of the proposed framework, the SSL module can identify the unlabeled data for learning the retinal image, and another one is the pretext tasks, which includes

- Rotation prediction—it will be used to rotate the retinal image at different angles.
- Puzzle Prediction—the retinal images reconstruct the spatial arrangement of the image patches.
- Patch Reconstruction is the process of dividing an image into patches, which are masked from the spatial representation of the image (Figure 3).

**Figure 2:**
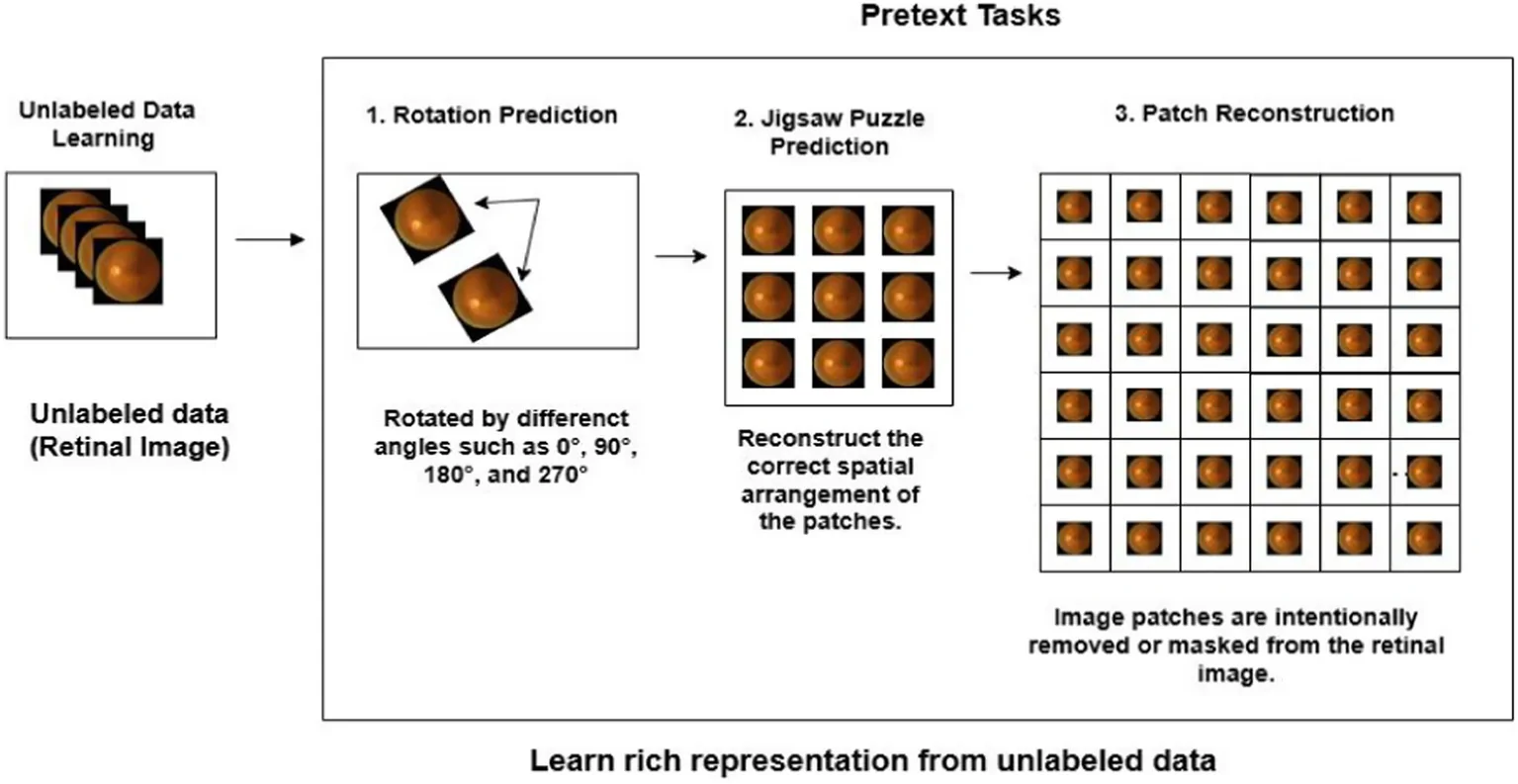
Self-supervised learning (SSL) module.

**Figure 3:**
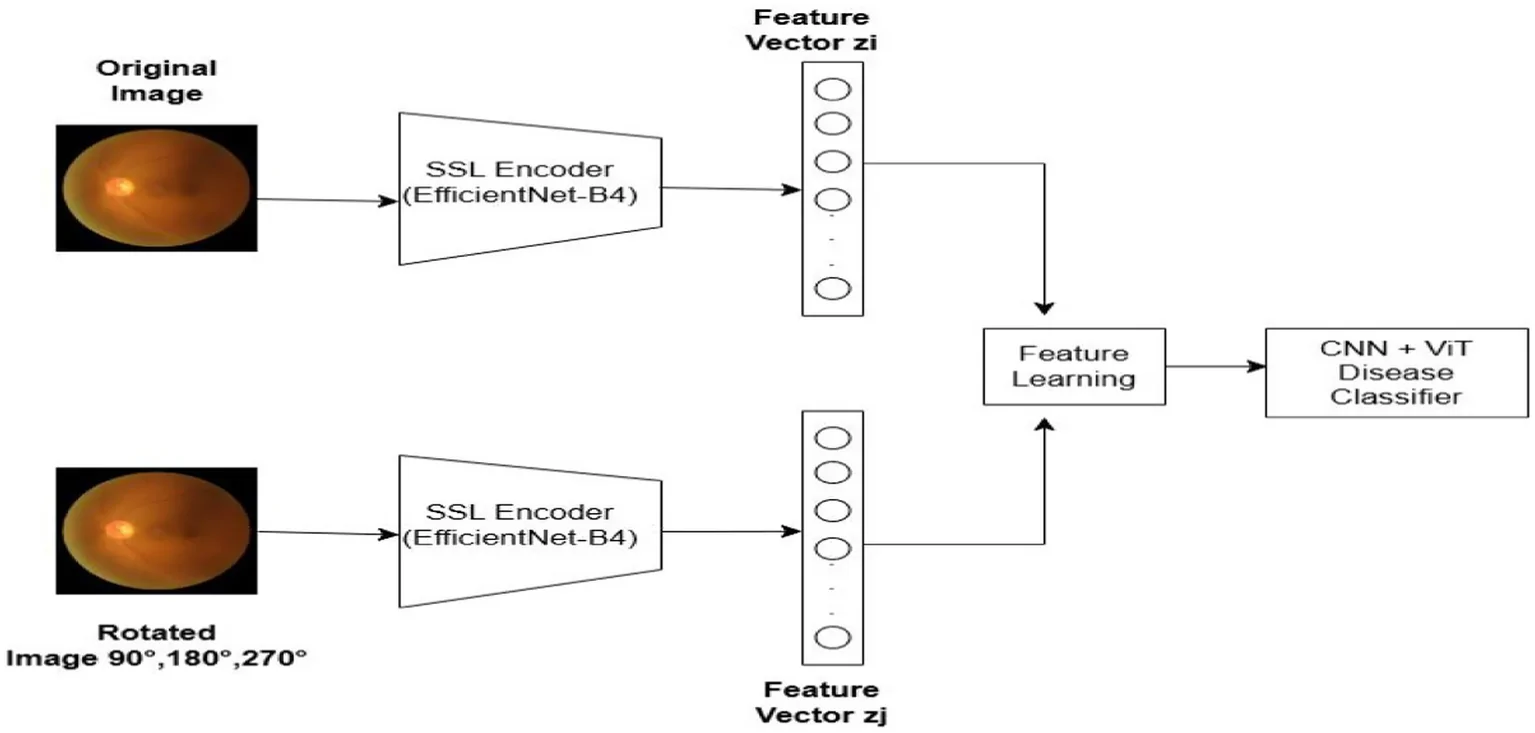
Retinal image rotation for Self-Supervised Learning (SSL) using the CNN EfficientNet-B4 encoder.

Self-Supervised Learning is employed before supervised training. The SSL module uses a large collection of unlabeled retinal images with BCEWithLogitsLoss, using a SimCLR method, and learns to perform useful complete pretext tasks for retinal images, such as Contrastive Learning. Different augmented versions of the same image are shown as positive pairs, while dissimilar images are shown as negative pairs. The contrastive loss is given by Equation 2, where the temperature parameter π is used as shown below,


$$\text{SSLVNet}(L_{\mathrm{SSL}})=-\log(\frac{\exp(\mathrm{Sa}m_{\mathrm{pos}}/\pi )}{\sum_{a=1}^{n}\exp(\mathrm{Sa}m_{\mathrm{fea}}/\pi )})$$ (2)

Where,

-
$\text{SSLVNet}(L_{\mathrm{SSL}})$ = Self-Supervised learning loss
-
$\mathrm{Sa}m_{\mathrm{pos}}$ = Positive pair for the input data
-
$\mathrm{Sa}m_{\mathrm{fea}}$ = Feature pair for the input data
-
π = Temperature parameter for the input data

### 3.3 Dual backbone feature extraction

Figure 4, Dual function is the third step, which is double-backbone feature extraction to capture both local and global retinal information; the architecture uses two parallel feature-removal systems. It can involve learning the retinal patterns from the fundus images.

**Figure 4:**
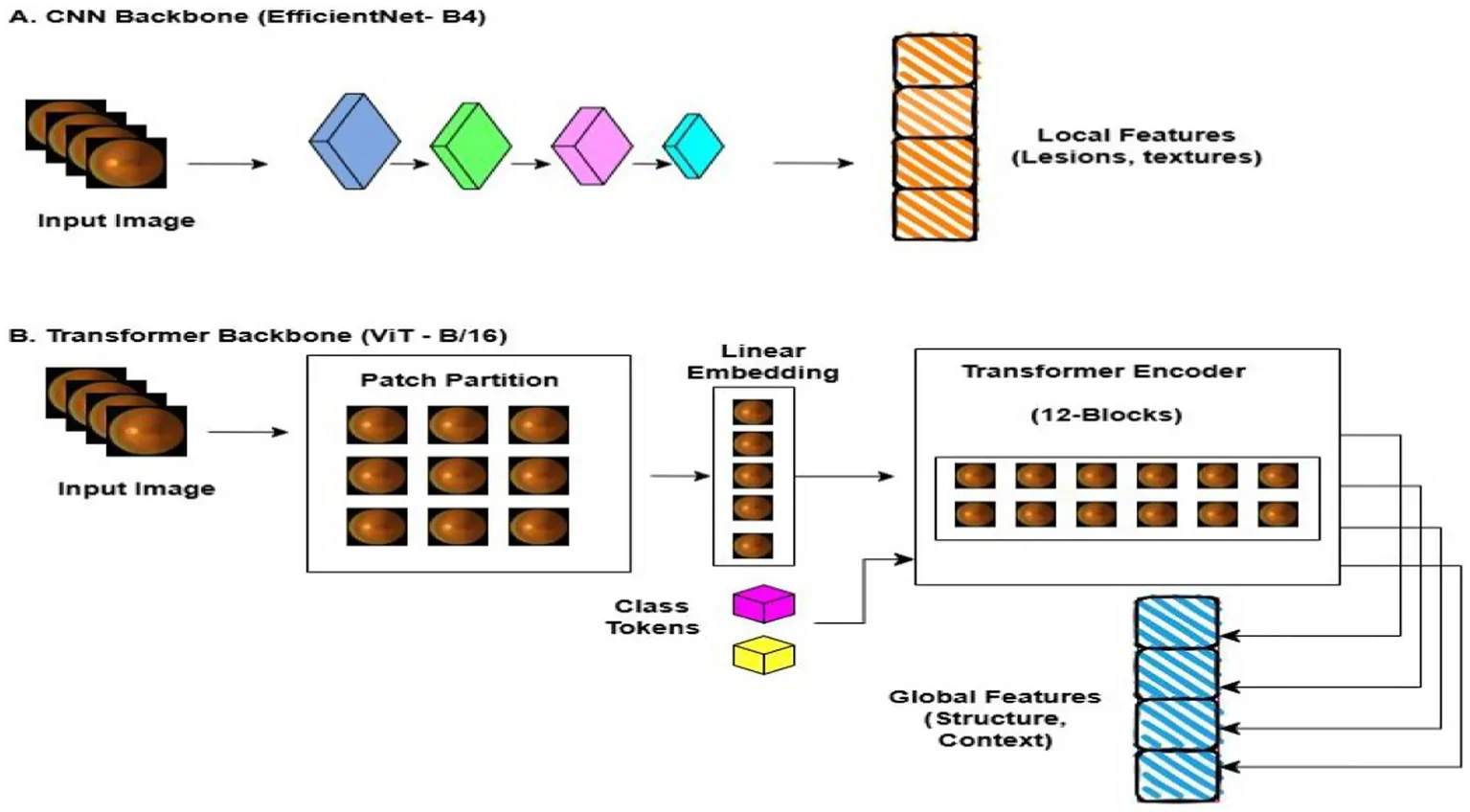
Dual function for feature extraction in retinal image. **(A)** CNN backbone (EfficientNet-B4) for local feature extraction (lesions, textures); **(B)** Transformer backbone (ViT-B/16) for global feature extraction (structure, context).

#### 3.3.1 CNN backbone (EfficientNet-B4)

Figure 5 shows the EfficientNet-B4 working as a CNN image classifier, especially designed for image processing. CNN will automatically detect feature extraction from the retinal image. In Convolutional Layers - The input image passes data from the convolutional layer, which can detect edges, textures, and patterns, and the convolution operation is defined in Equation 3, which computes the output response using the input image and kernel filters. Initially, a 9 × 9 format image is generated, which will cover the 0-black representation. Then each 3 × 3 matrix is converted to the one output matrix for using a 3 × 3 kernel matrix. Convolution with horizontal and vertical strides = 1. The pooling layer reduces the image size or spatial size. The max pooling uses the feature map to generate a 2 × 2 matrix, and the output is the max-pooled feature map value. It uses the pooled feature map to convert the Convoluted image to a max pooling image. The avg. pooling uses the feature map to generate a 2 × 2 matrix, whose output is the avg-pooled feature map value. It uses the pooled feature map to convert the Convoluted image to an average pooling image. The Fully Connected Layer performs the final classification and prediction of the output class. CNN backbone for local structures to extract from fundus images. CNNs are highly effective in detecting local image features. The input picture size is 224 × 224 × 3, and the image is processed for the preprocessing stage, such as resizing, normalization, and data augmentation. The convolution operation is the first stage process for EfficientNet-B4, which applies the convolution filters to the retinal fundus image.


$$f_{o/p}(f_{1},f_{2})=\sum_{i}\sum_{j}Q_{i}(f_{1}-x,f_{2}-y)S_{k}(u,v)$$ (3)

**Figure 5:**
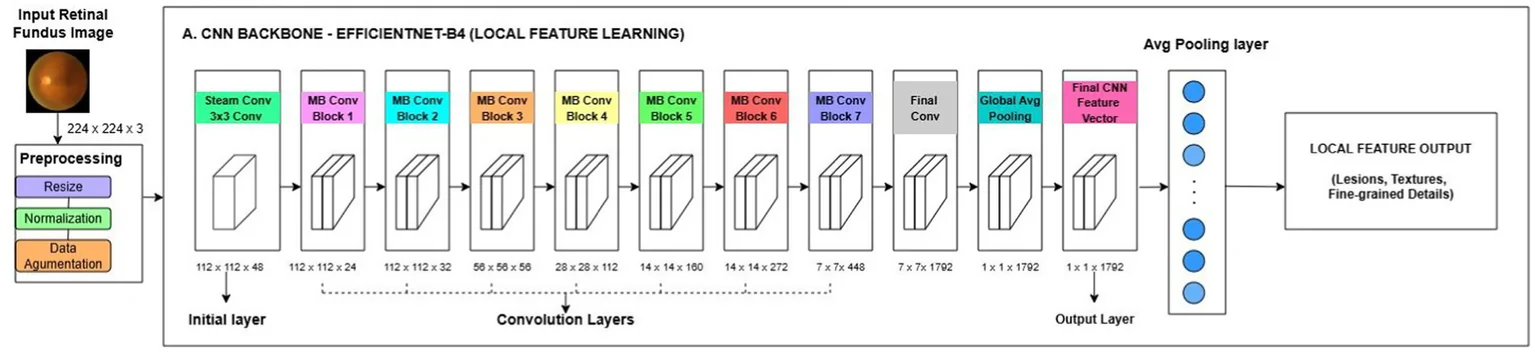
CNN backbone (EfficientNet-B4).

Where,

-
$f_{o/p}$ = Output response
-
$Q_{i}$ = Input for the retinal eye image
-
$S_{k}$ = Retinal eye kernel

The second stage MBConv Block is the process of stream convolution, which can be an 112 × 112 × 3 input layer to a 1 × 1 × 1,792 output layer. The layers are working in a block-to-block manner. So, the input layers are extended to increase the feature image. Applying filters to all the blocks, each block remains managed self-sufficiently. The output CNN feature vector is signified as shown in Equation 4,


$$\text{SSLVNe}t_{f(\mathrm{CNN})}=[f1,f2,f3,f4,\ldots \mathrm{fn}]$$ (4)

Where,

-
$\text{SSLVNe}t_{f(\mathrm{CNN})}$ = It signifies the retinal fundus image information

#### 3.3.2 Vision transformer (ViT-B/16)

Figure 6 captures the global feature information from the retinal fundus images. It uses a self-attention mechanism. It will be used for long-range needs and spatial relations. The input retinal fundus image is processed in the preprocessing stage, such as resizing, normalization, and data augmentation. The ViT does not process the full image directly. The image will be a patch partition; the patch size is 16 × 16. Every patch is converted into a feature vector. The image will be converted into multiple blocks (small blocks), and it can be processed through linear projection and positional encoding. Each patch is converted into an embedding vector. Each retinal image patch connects with every other image patch, as computed in Equation 5 using the self-attention mechanism,


$$\text{SSLVNe}t_{A}(f_{Q},f_{K},f_{V})=\text{Softma}x_{i}(\frac{f_{Q}{f_{K}}^{T}}{\sqrt{d}})f_{V\;\;}$$ (5)

**Figure 6:**
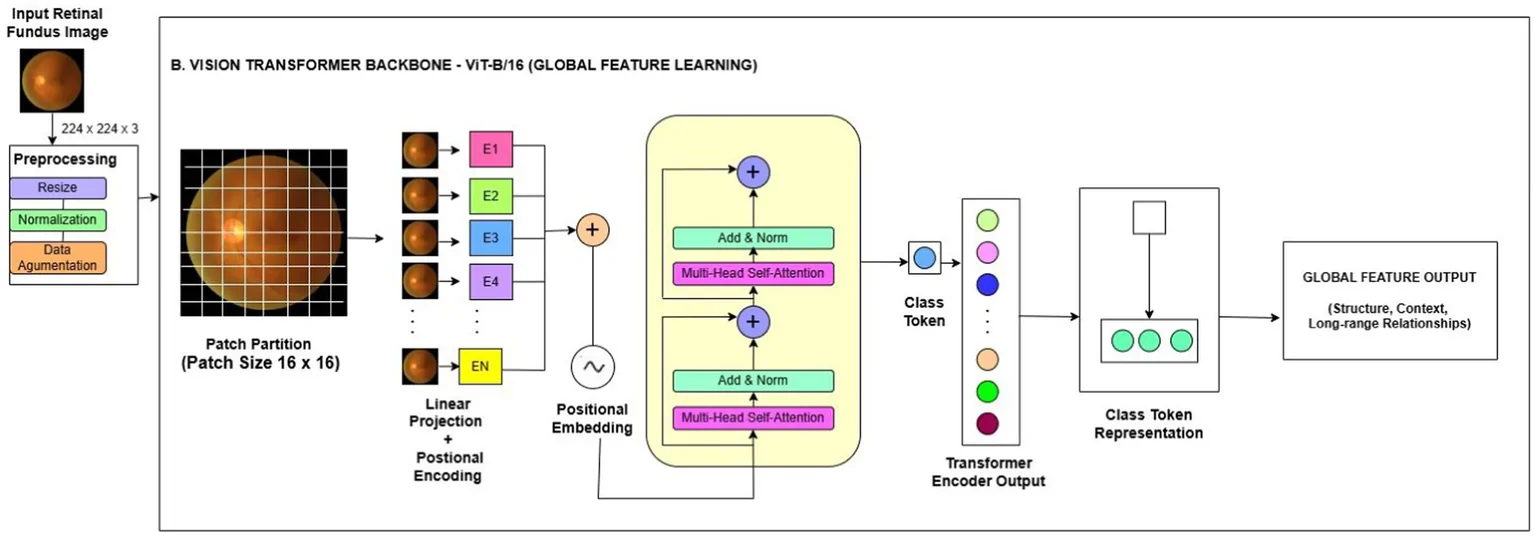
CNN backbone – a Vision Transformer (ViT-B/16).

Where,

-
$\text{SSLVNe}t_{A}$ = Self-attention output for using query, key, value
-
$f_{Q}$ = query (Q) matrices of each patch in the input
-
$f_{K}$ = key (K) matrices of each patch in the input
-
$f_{V}$ = value (V) matrices of each patch in the input
-
d = dimension
-
$\text{Softma}x_{i}$ = It is used to create the attention weights

The Vision Transformer input has patch 1 and patch 2, which include the 512-dimensional embedding represented as 1×512. The 64-dimensional queries represent the current patch as 1×64, including $q_{1}$ and $q_{2}$ . These query representations are denoted by $f_{Q}$ . The 64-dimensional keys represent the content of each patch (like a label or descriptor) as 1×64, including *k*_1_ and *k*_2_. These key representations are denoted by *f*_k_. The 64-dimensional values hold the actual information of each patch to be aggregated as 1×64, including $v_{1}$ and $v_{2}$ . These value representations are denoted by $f_{v}.$

### 3.4 Feature fusion module

The fourth step features a fusion module that is developed to combine both local and global features, including different feature extractions. The fused feature image is obtained using Equation 6,


$$f_{\text{fusion}}=\text{Concat}(f0_{\mathrm{CNN}},f1_{\mathrm{ViT}})$$ (6)

The fusion layer learns relationships between image features. This step makes a complete representation of retinal disease characteristics. Initially, the retinal fundus image is processed using EfficientNet-B4—local features and Vision Transformer B/16 global features are included for the proposed work.

### 3.5 Hierarchical transformer module

The fifth step is the hierarchical transformer module, which has the feature data from different modules. The encoding is all matching in structure. The Multi-Head Self-Attention layer, which includes multiple attention heads that focus on different retinal data, is defined in Equation 7,


$$\text{SSLVNe}t_{\mathrm{MHA}}(f_{Q},f_{K},f_{V})=\text{Conca}t_{\mathrm{ope}}(\begin{array}{l} f_{H_{1}},f_{H_{2}},\\ f_{H_{3}},\ldots ,f_{H_{h}} \end{array})WM_{o}$$ (7)

Where,

-
$f_{H_{1}},f_{H_{2}},f_{H_{3}},\ldots ,f_{H_{h}}$ = Multiple attention heads for the outputs
-
$\text{Conca}t_{\mathrm{ope}}$ = Concatenation for the output operation
-
$WM_{o}$ = weights of the output matrix

Feed Forward Network is refining the extracted images, and layer normalization is improving the convergence and training stability.

### 3.6 Lesion graph network (LGN)

The sixth step, lesion graph network, which is frequently considered in terms of relationships among multiple lesions. Instead of treating lesions in isolation, the proposed framework models lesion communications as happening using graph structures. The Graph Attention Network informs node features as shown in Equation 8,


$$\text{SSLVNe}t_{\text{out}}=\text{ReLU}\;(\sum_{a=1}^{N}W_{i}Rr_{i})$$ (8)

Where,

-
$\text{SSLVNe}t_{\text{out}}$ = Output
-
$Rr_{i}$ = Feature of the neighboring retinal region/lesion
-
$W_{i}$ = Weight showing the importance of the neighboring region
-
N = Neighboring retinal regions/lesions

### 3.7 Multi-label classification head

The seventh step is a module that focuses on developing an AI-based hybrid deep learning framework for automating all retinal diseases. It may contain multiple diseases instantaneously. To identify the final prediction of the class. The final output layer uses Sigmoid because multiple diseases can be predicted at the same time. The sigmoid activation function used is given in Equation 9,


$$\sigma (f_{z}x)=\frac{f_{1}}{f_{1}+f_{a}e^{-x}}$$ (9)

Where,

-
$\sigma (f_{z}x)$ = Output sigmoid
-
$f_{z}x$ = Input value from the previous layer
-
$f_{a}e$ = Constant of the input
-
$f_{1}$ = Input value of the current layer

### 3.8 Prediction uncertainty estimation

The uncertainty score is computed using Equation 10,


$$\text{SSLVNe}t_{U}=\frac{1}{n}\;\sum_{a=1}^{n}{\left({\mathrm{Pr}}_{i}-{\mathrm{Pr}}_{m}\right)}^{2}$$ (10)

Where,

-
$\text{SSLVNe}t_{U}$ = Uncertainty score of the model prediction
-
n = Number of the predictions considered
-
${\mathrm{Pr}}_{i}$ = Probability of the individual prediction
-
${\mathrm{Pr}}_{m}$ = Mean probability of the predictions

The eighth step is ambiguity estimation for the required confidence data to add to predictions. This process helps to predict the probability distribution of each class. It can predict the confidence score for each class, for comparison of all scores, and which score is best, which helps for the final prediction.

### 3.9 Explainability module

The ninth step uses the heatmap (Grad-CAM) to identify the most important places in the retinal image and the less important places in the retinal image.

### 3.10 Final output function

The output of the proposed model includes the predicted disease for multi-classes, a confidence score for each class, and a heatmap conception, total loss combining SSL and classification loss is given in Equation 11,


$$f_{o/p}=w_{1}f_{\mathrm{fea}}+w_{2}f_{\text{class}}$$ (11)

Where,

-
$f_{o/p}$ = Total loss of the model
-
$w_{1}f_{\mathrm{fea}}$ = learning loss
-
$w_{2}f_{\text{class}}$ = class loss

## 4 Experimental setup

The hybrid multi-label proposed framework for retinal eye disease classification was implemented and evaluated using the ODIR-5k retinal eye fundus image dataset in Google Colab. It can cover the eight types of disease classes from the retinal images. Before training, all the category images were resized to 224 × 224 pixels, and the input data were normalized to ensure consistency. The proposed model combines Self-Supervised Learning, a CNN backbone for EfficientNet-B4, and a Vision Transformer (ViT-B/16). Multi-model fusion image features for feature extraction are based on CNN backbones, and finally classified multi-label retinal eye diseases are trained for 20 epochs. The ODIR-5K dataset has an imbalanced class; therefore, loss functions (Weighted BCEWithLogitsLoss) are used during training to give greater importance to minority classes. Table 2 provides the entire experimental setup.

**Table 2:** Experimental details.

Sl. no	Parameter	Value
1	Random seed	42
2	Train size	0.7
3	Validation size	0.15
4	Test size	0.15
5	Image size	224 × 224
6	Batch size	16
7	Num workers	2
8	Optimizer	AdamW
9	Learning rate	0.0001
10	Weight decay	0.01
11	Loss function	Weighted BCEWithLogitsLoss
12	Decision threshold	0.5
13	Maximum epochs	20
14	Early stopping patience	5
15	Early stopping metric	val_macro_f1
16	SSL epochs	5
17	SSL temperature	0.2
18	Bootstrap repeats	1000
19	Alpha (significance level)	0.05
20	Mixed precision (AMP)	True

### 4.1 Performance evaluation metrics

1) Accuracy is calculated using Equation 12,

$$\text{SSLVne}t_{\mathrm{Acc}}=\frac{t_{\mathrm{pos}\_i}+t_{\mathrm{neg}\_j}}{t_{\mathrm{pos}\_i}+t_{\mathrm{neg}\_j}+f_{\mathrm{pos}\_i}+f_{\mathrm{neg}\_j}}$$ (12)

2) Precision is calculated using Equation 13,

$$\mathrm{SSL}V_{\mathrm{Pre}}=\frac{t_{\mathrm{pos}}}{t_{\mathrm{pos}}+f_{\mathrm{pos}}}$$ (13)

3) Sensitivity (Recall) is calculated using Equation 14,

$$\text{SSLVne}t_{\mathrm{Sen}\_\text{recall}}=\frac{t_{\mathrm{pos}}}{t_{\mathrm{pos}}+f_{\mathrm{neg}}}$$ (14)

4) Intersection over Union (IoU) is calculated using Equation 15,

$$\text{SSLVne}t_{\mathrm{IOU}}=\frac{t_{\mathrm{pos}}}{t_{\mathrm{pos}}+f_{\mathrm{pos}}+f_{\mathrm{neg}}}$$ (15)

5) The Dice score is calculated using Equation 16,

$$\mathrm{SSL}V_{\text{Dice}}=\frac{2\times t_{\mathrm{pos}}}{(2\times t_{\mathrm{pos}})+f_{\mathrm{pos}}+f_{\mathrm{neg}}}$$ (16)

6) Exact Match Accuracy is calculated using Equation 17,

$$\mathrm{SSL}V_{EM_{\mathrm{Acc}}}=\frac{n_{\mathrm{ex}\_\mathrm{acc}}}{n_{\mathrm{tol}\_\mathrm{sam}}}$$ (17)

7) Hamming Accuracy is calculated using Equation 18,

$$\text{SSLVne}t_{H_{\mathrm{Acc}}}=\frac{1}{m\times n}\sum_{a=1}^{m}\sum_{b=1}^{n}I_{f}\;(C_{\mathrm{ab}}=D_{\mathrm{ab}})$$ (18)

8) The F1-score is calculated using Equation 19,

$$\text{SSLVne}t_{\left(F1-\text{score}\right)}=2\times \frac{\mathrm{SSL}V_{\mathrm{Pre}}\times \mathrm{SSL}V_{\mathrm{sen}\_\text{recall}}}{\mathrm{SSL}V_{\mathrm{pre}}+\mathrm{SSL}V_{\mathrm{sen}\_\text{recall}}}$$ (19)

9) The true positive rate used for the ROC curve is calculated using Equation 20,

$$\text{SSLVne}t_{\left(T_{\text{posR}}\right)}=\frac{t_{\mathrm{pos}}}{t_{\mathrm{pos}}+f_{\mathrm{neg}}}$$ (20)

10) The false positive rate is calculated using Equation 21,

$$\text{SSLVne}t_{\left(F_{\text{posR}}\right)}=\frac{f_{\mathrm{pos}}}{f_{\mathrm{pos}}+t_{\mathrm{neg}}}$$ (21)

11) The confusion matrix structure is given in Equation 22,

$$\text{SSLVne}t_{\mathrm{CM}}=[\begin{array}{cc} t_{\mathrm{pos}} & f_{\mathrm{pos}}\\ f_{\mathrm{neg}} & t_{\mathrm{neg}} \end{array}]$$ (22)

12) The Grad-CAM heatmap is computed using Equation 23,

$$\text{SSLVne}t_{\begin{array}{c} \mathrm{gra}d_{\mathrm{cam}} \end{array}}=\mathrm{ReL}U_{a}(\sum_{j=1}^{i}\propto_{i}A_{j})$$ (23)

## 5 Results and discussion

Figure 7 shows the training loss reduced from 0.3304 to 0.0154 in the retinal image, and the validation loss reduced from 0.2979 to 0.1497 in the retinal image. The small changes in the train loss and validation loss are because the curve suggests decent generation and limited overfitting. This image shows that the model can learn the retinal eye image features in detail during training. The validation loss remains stable during the training process. Epoch 10 has the lowest validation loss of 0.1237.

**Figure 7:**
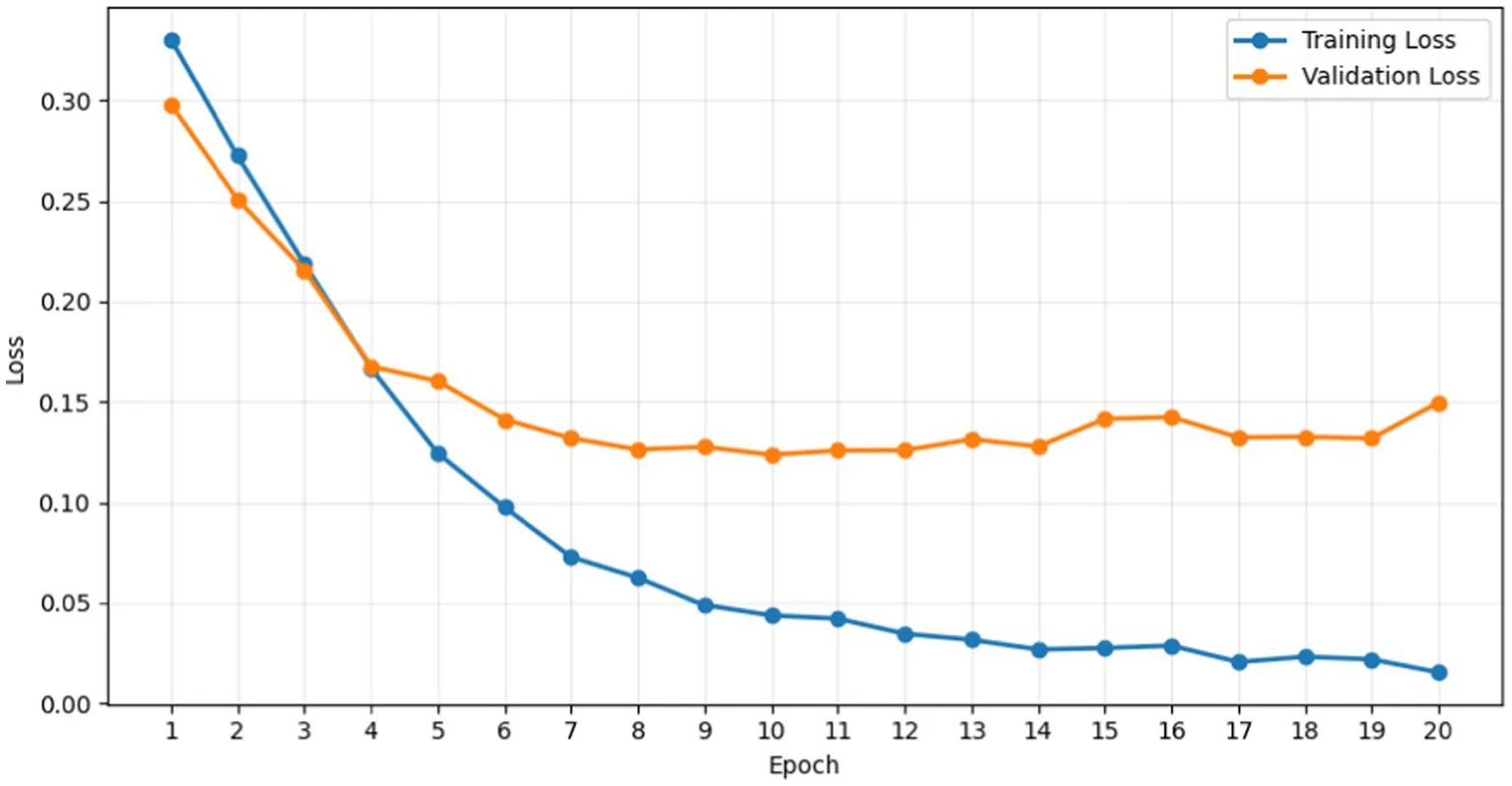
Training and validation loss.

Figure 8 shows the model performance of the Macro F1-score over 20 training epochs. The score has increased consistently from 0.3019 to 0.9036. The highest epoch is achieved at epoch 16.

**Figure 8:**
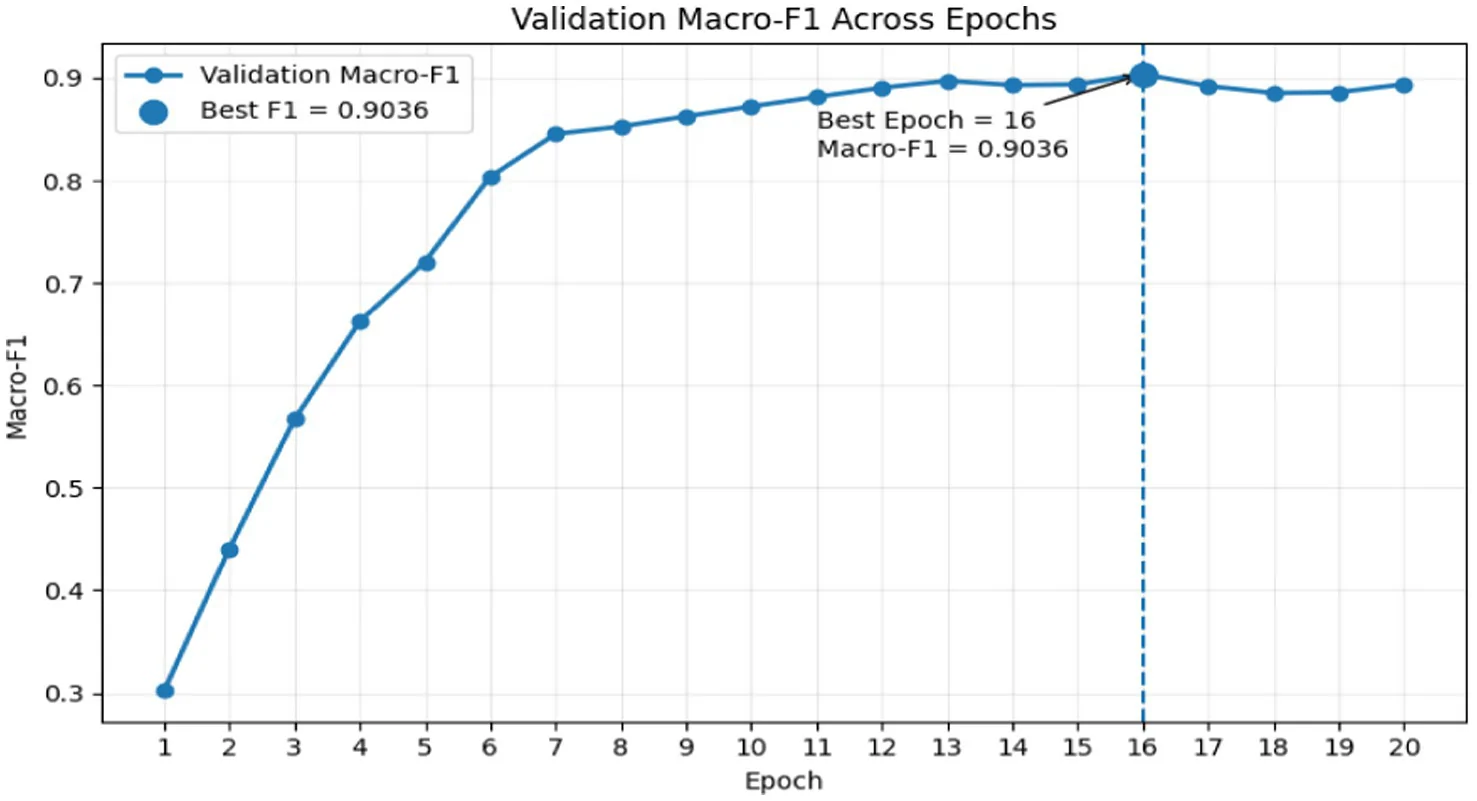
Macro F1-score.

Figure 9 shows that the model performance displays good performance during the training process. It indicates that all three metrics improve progressively as the number of epochs increases from 24.78 to 84.68%. The highest Exact Match Accuracy of 85.73% was reached at Epoch 17. The Hamming accuracy has reached 96.92%. The F1-score improves from 30.29 to 90.36%. Validation Macro F1-score was used as the main criterion for selecting the final model checkpoint. The validation loss and Exact Match Accuracy were reported as additional performance measures, but they were not used for model selection. The highest Validation Macro-F1 was obtained at epoch 16, and the checkpoint from epoch 16 was selected as the final model. The test set was not involved in selecting the epoch or tuning the model.

**Figure 9:**
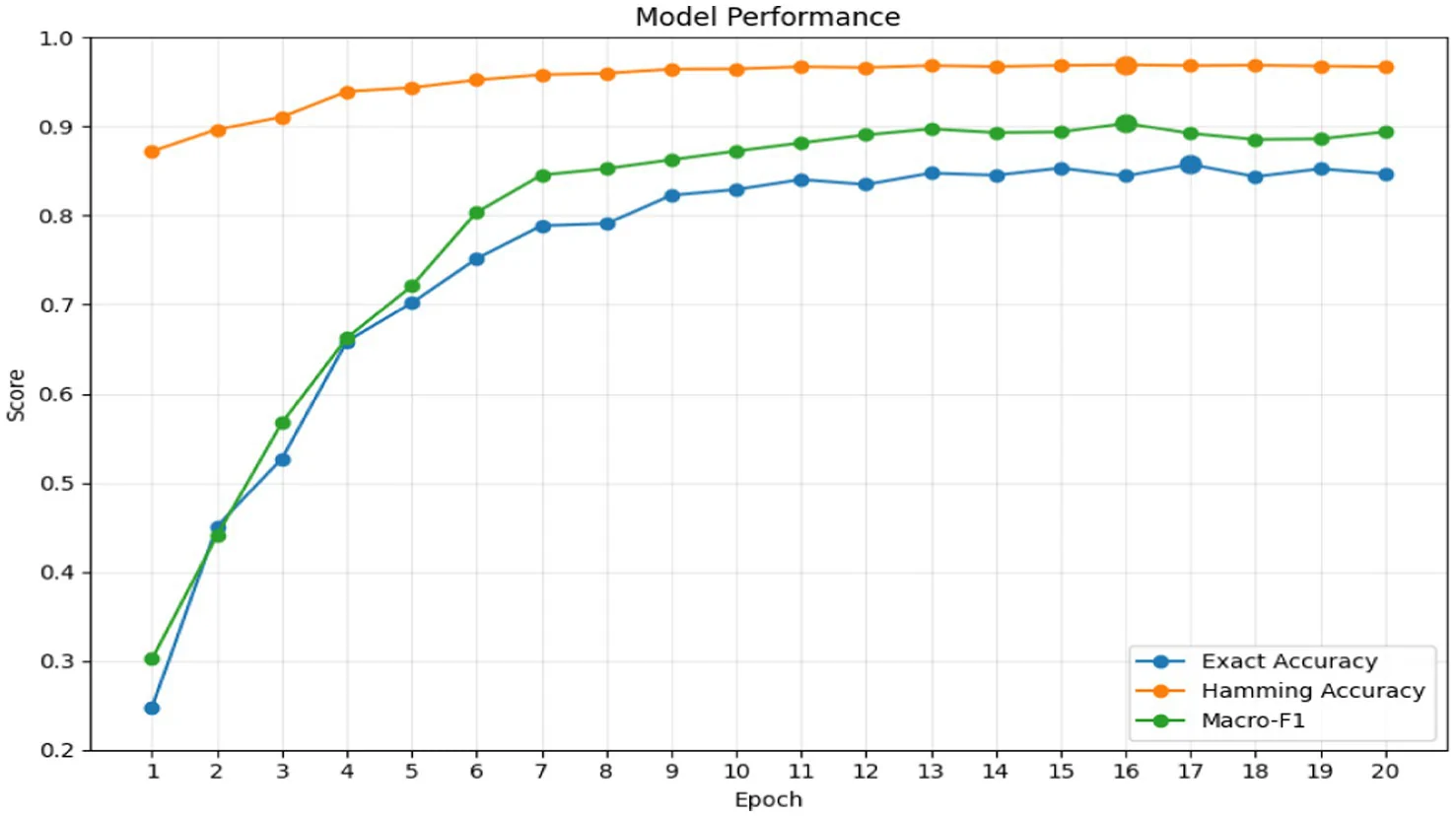
Exact accuracy, Hamming accuracy, and F1-score graph.

Table 3 shows that the proposed SSLVNet performs the best among the other baseline models. The highest F1 score achieved by SSLVNet is 0.9036, when compared to the other models. The proposed model has reached the highest Exact Match Accuracy of 0.8444, Hamming Accuracy of 0.9692, and Macro F1-score of 0.9036.

**Table 3:** Comparison of the proposed SSLVNet with baseline models.

Methods	Exact match acc	Hamming acc	Macro F1
Proposed SSLVNet	0.8444	0.9692	0.9036
EfficientNet-B4 (CNN-only)	0.7273	0.9501	0.8134
ViT-B/16 (ViT-only)	0.7169	0.9442	0.7991
CNN + ViT fusion (no SSL/attention/graph)	0.7081	0.9469	0.7549

Table 4 shows the ablation study of the proposed SSLVNet model, that reached the best performance among other methods. Methods without the CNN branch: the F1-score is 0.6353, without the ViT branch is 0.7895, without the Lesion Graph feature is 0.7577, without Lesion Attention is 0.7930, and the proposed SSLVNet reached the highest F1-score of 0.9036.

**Table 4:** Ablation study.

Methods	Exact match acc	Hamming acc	Macro F1-score
Full model (Proposed SSLVNet)	0.8444	0.9692	0.9036
w/o lesion attention	0.7017	0.9446	0.7930
w/o lesion graph feature	0.7041	0.9424	0.7577
w/o ViT branch	0.7281	0.9500	0.7895
w/o CNN branch	0.5485	0.9159	0.6353

Table 5 shows the computational analysis for the proposed SSLVNet, with the highest computational requirements compared to the other baseline models, while requiring fewer parameters, FLOPs, model size, and peak GPU memory than the CNN + ViT fusion model.

**Table 5:** Computational complexity and efficiency comparison of the proposed models.

Model	Parameters (M)	Trainable params (M)	MACs (G)	FLOPs (G)	Model size (MB)	Latency (ms/img)	Throughput (img/s)	Peak GPU memory (MB)
Proposed SSLVNet	104.729	104.729	18.348	36.695	390.508	21.721	46.038	6206.654
EfficientNet-B4 (CNN-only)	24.561	24.561	4.133	8.265	93.694	6.16	162.329	6166.724
ViT-B/16 (ViT-only)	85.805	85.805	16.848	33.695	327.319	14.448	69.216	6381.959
CNN + ViT fusion (no SSL/attention/graph)	110.753	110.753	20.981	41.962	422.49	19.421	51.492	6498.793

Figure 10 shows the disease-wise test performance metrics of the proposed model, including Sensitivity, Specificity, Precision, IoU, Dice score, and Accuracy for retinal eye disease classes. The model achieved good performance for all retinal disease classes. The results vary across the different diseases. Myopia (M) has the best performance, followed by Cataract (C), which shows very high performance. Diabetic Retinopathy (D), Glaucoma (G), and AMD (A) show good and consistent results. Hypertension (H) has low Sensitivity (78.4%). Other (O) is the most challenging class, with lower IoU (67.4%) and Dice Score (80.6%). IoU and Dice scores for the multi-label classification are used to predict the true labels, not image pixels. Table 6 provides the mapping of code with keywords.

**Figure 10:**
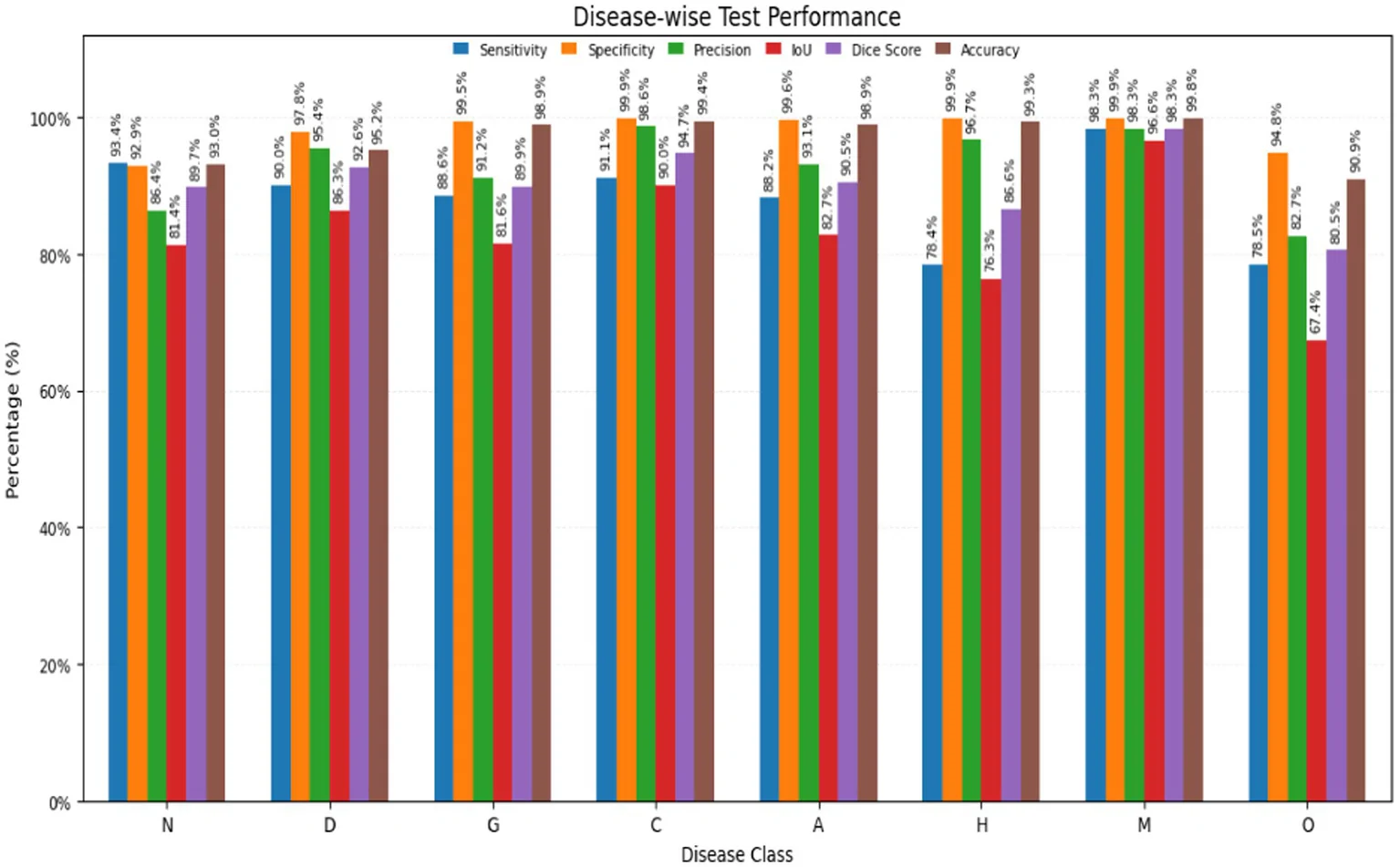
Disease-wise test performance metrics.

**Table 6:** ODIR-5K diagnostic keyword mapping.

Code	Disease	Diagnostic keywords
N	Normal	normal fundus
D	Diabetic retinopathy	diabetic retinopathy; no proliferative retinopathy
G	Glaucoma	glaucoma
C	Cataract	cataract
A	Age-related macular degeneration	age-related macular degeneration; macular degeneration
H	Hypertensive retinopathy	hypertensive retinopathy; hypertension
M	Myopia	myopic retinopathy; myopia; myopic maculopathy
O	Other	other diagnostic conditions not mapped to N, D, G, C, A, H, M

The false positive rate at the 0.5 threshold is depicted in Figure 11. The lowest FPR is observed for Cataract (C) at 0.0009, followed by Hypertension (H) and Myopia (M) at 0.0008. The highest FPR is Normal (N) at 0.0714, followed by Other (O) at 0.0516.

**Figure 11:**
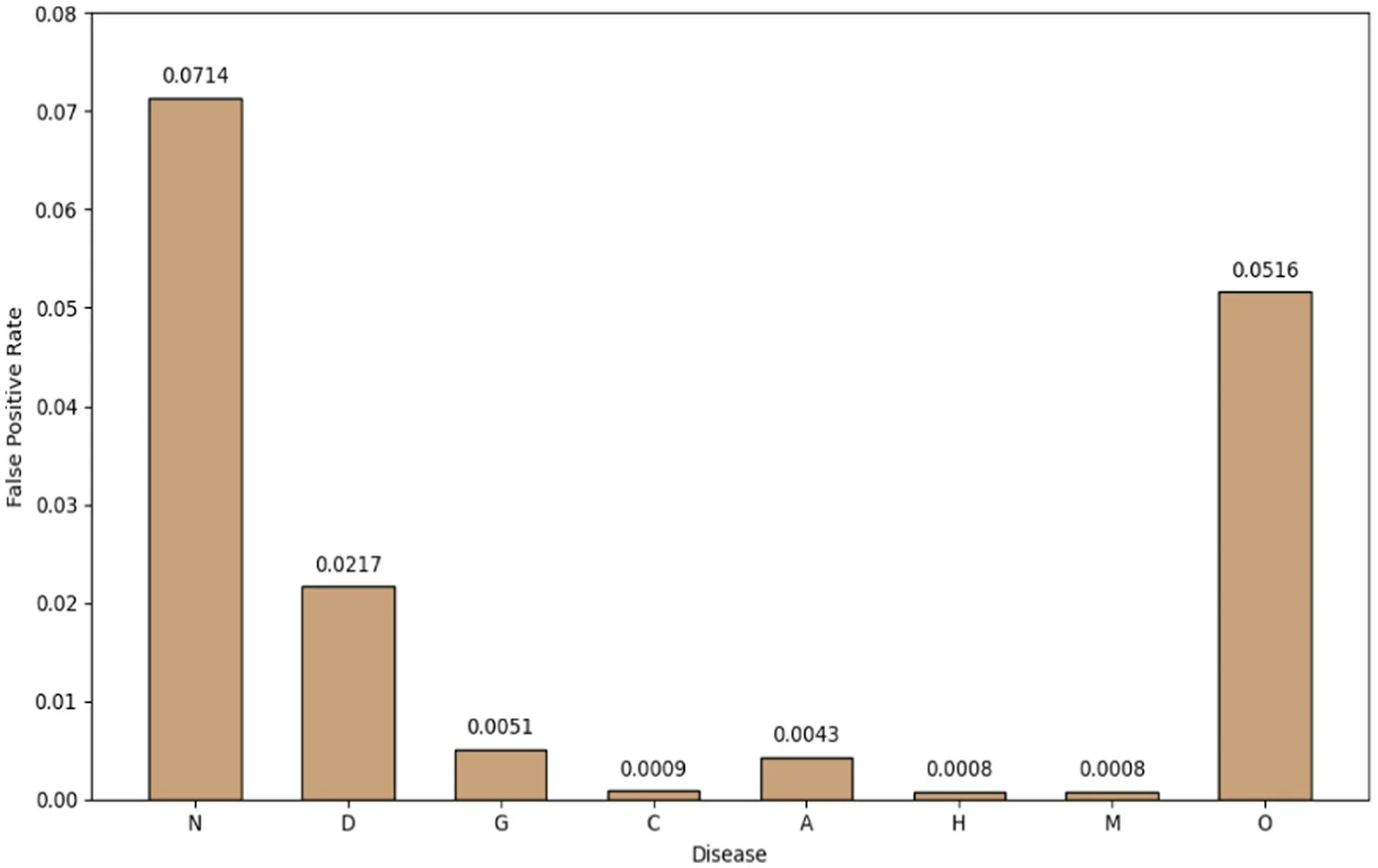
Per-class false positive rate at threshold 0.5.

The ROC Curve in Figure 12 shows the performance of the classification model. The *X*-axis indicates the False Positive Rate, and the *Y*-axis indicates the True Positive Rate. The high AUC value used for Myopia (M) is 0.99.

**Figure 12:**
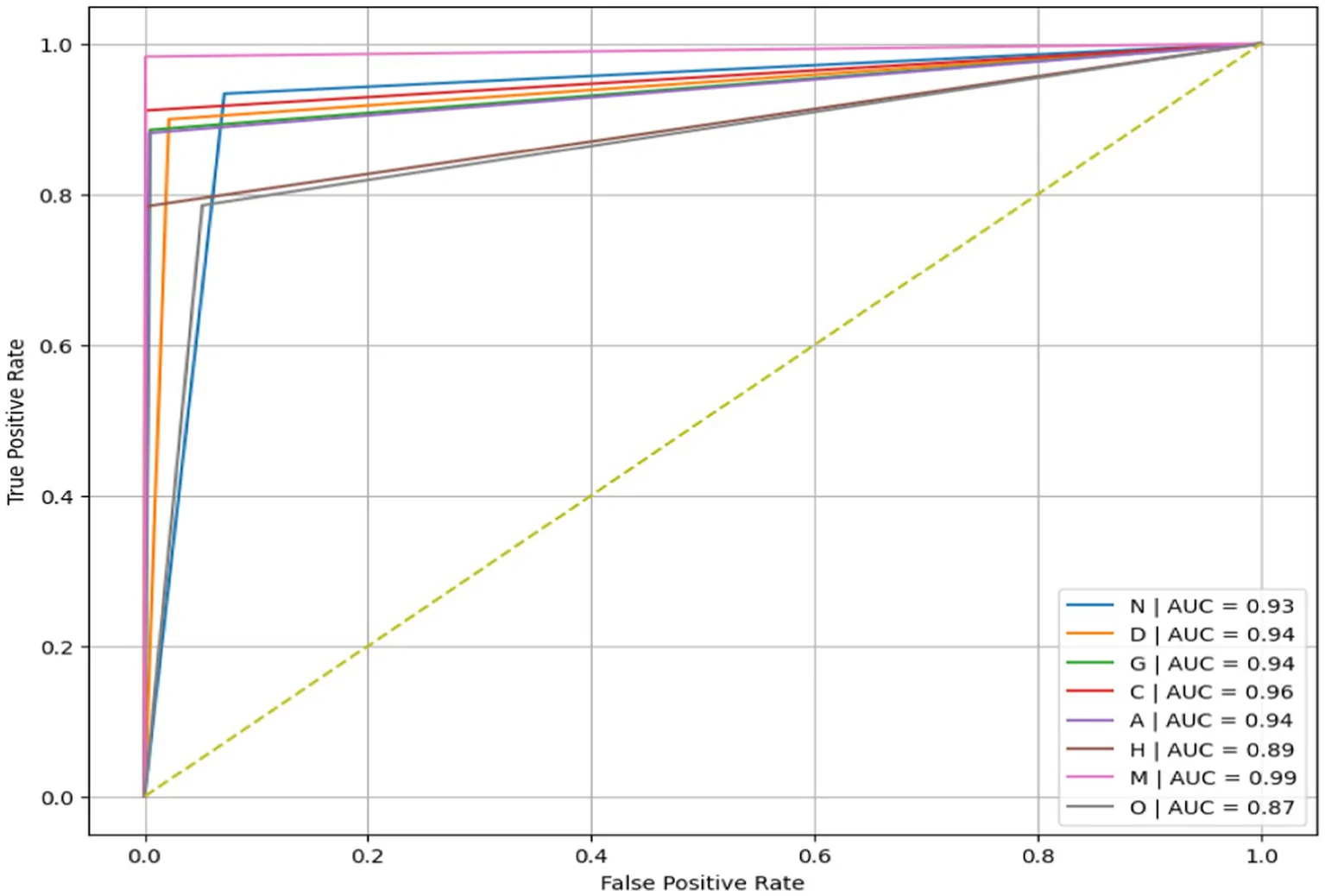
ROC curve.

The confusion matrix of the retinal eye image classification model indicates the correct classification of the retinal eye diseases for each class. Only a few data points are misclassified. Figure 13 represents the overall model and Figure 14 provides the label-wise classification of all eight disease categories. Most samples are correctly classified as retinal diseases. Only a few samples are misclassified.

**Figure 13:**
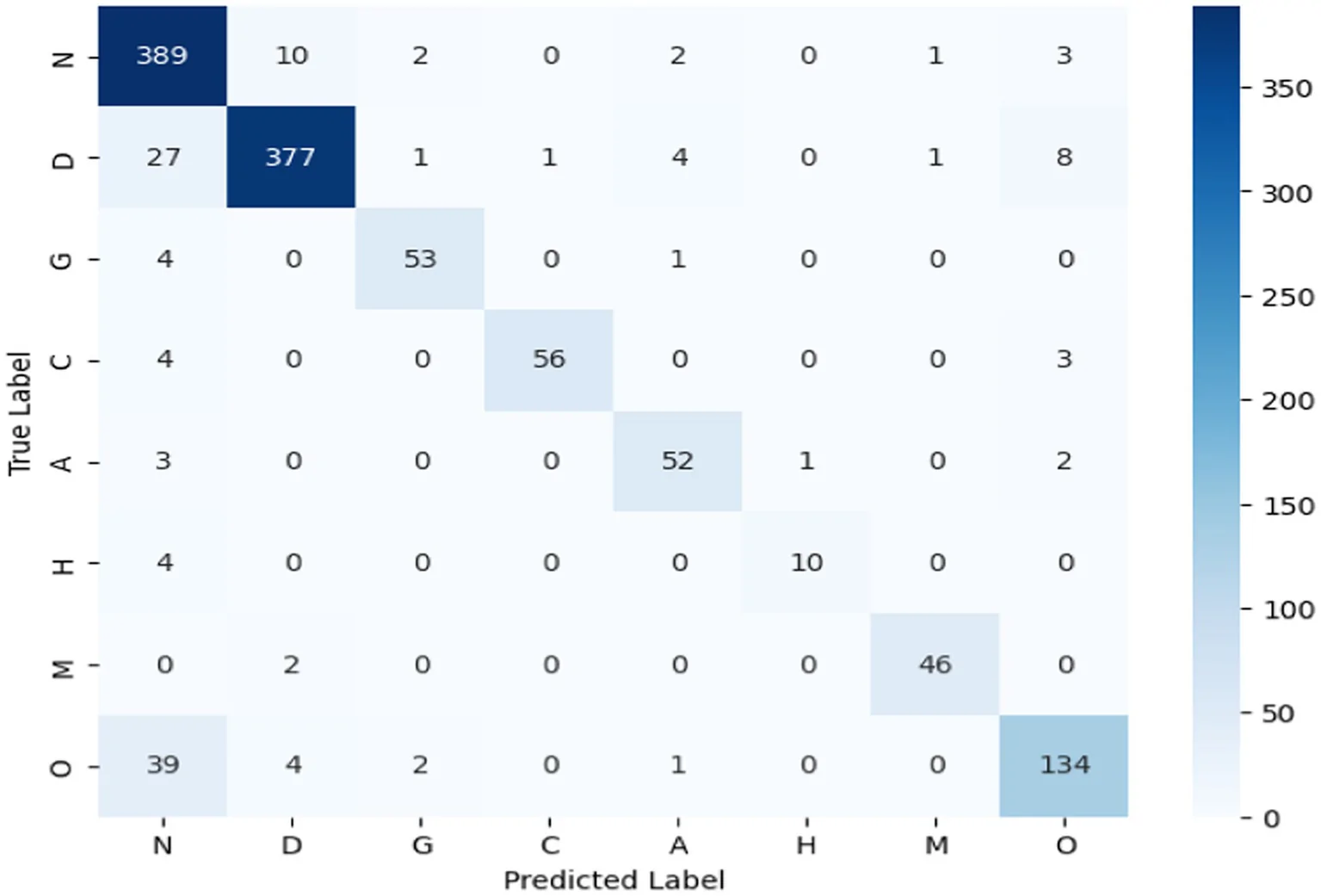
Confusion matrix.

**Figure 14:**
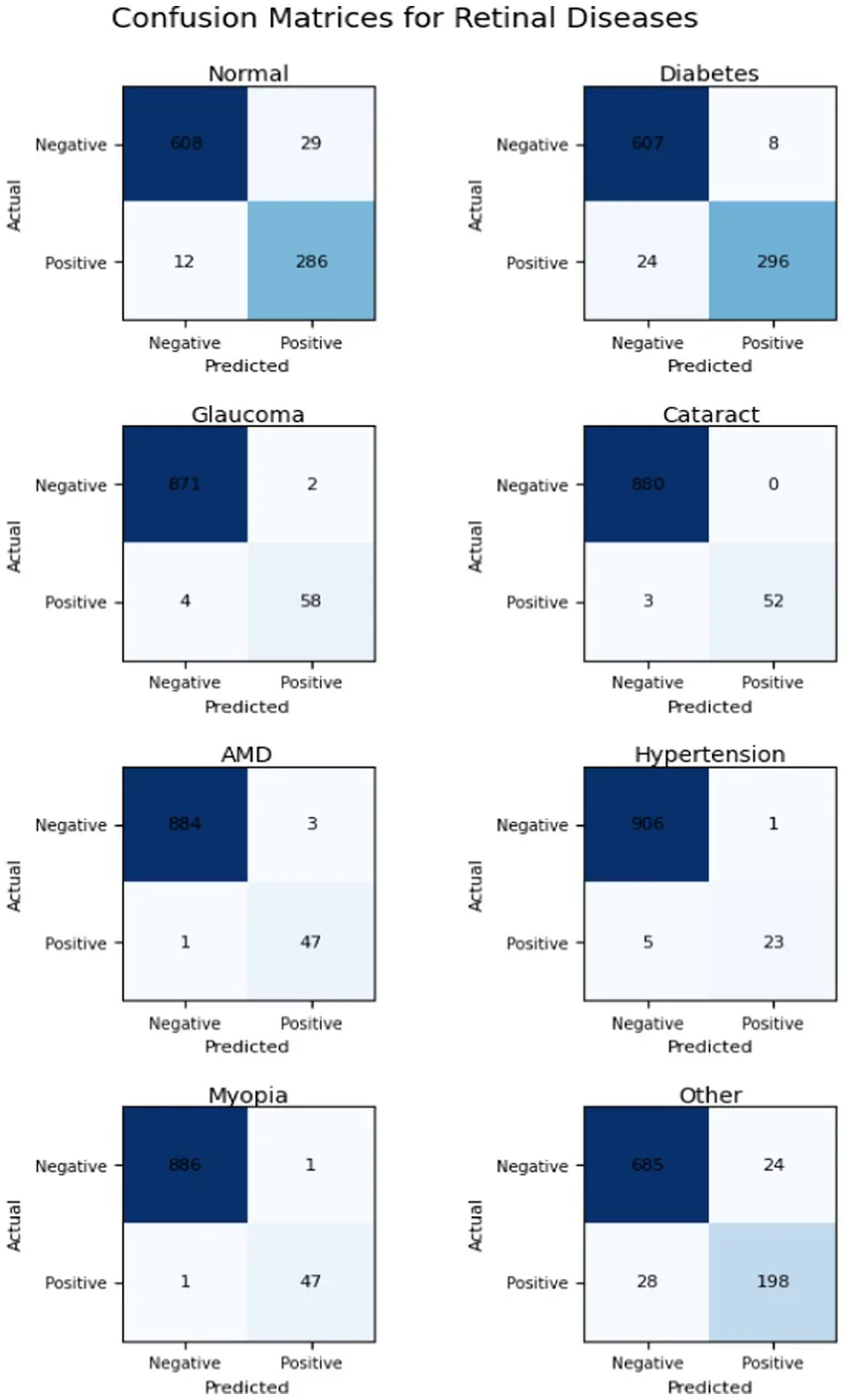
One-vs-rest confusion matrices for all labels.

Grad-CAM is used to identify the most important places in the retinal image that contribute most to image prediction. The image shows the eye regions highlighted in colors such as red, yellow, blue, and green. The red and yellow colors indicate the most important regions in the retinal image. Correct Predictions focus on relevant retinal regions. Incorrect Predictions focus on less consistent highlight areas. Grad-CAM Visualization images with their disease name, example type, true label, predicted label, and predicted probability are now provided. Corrected positive, false positive, and false negative cases are also clearly indicated. These details have been added to make it easier to understand the relationship between the model prediction and the corresponding Grad-CAM visualization. Figure 15 shows the Grad-CAM images for correct and incorrect predictions of all the retinal eye diseases.

**Figure 15:**
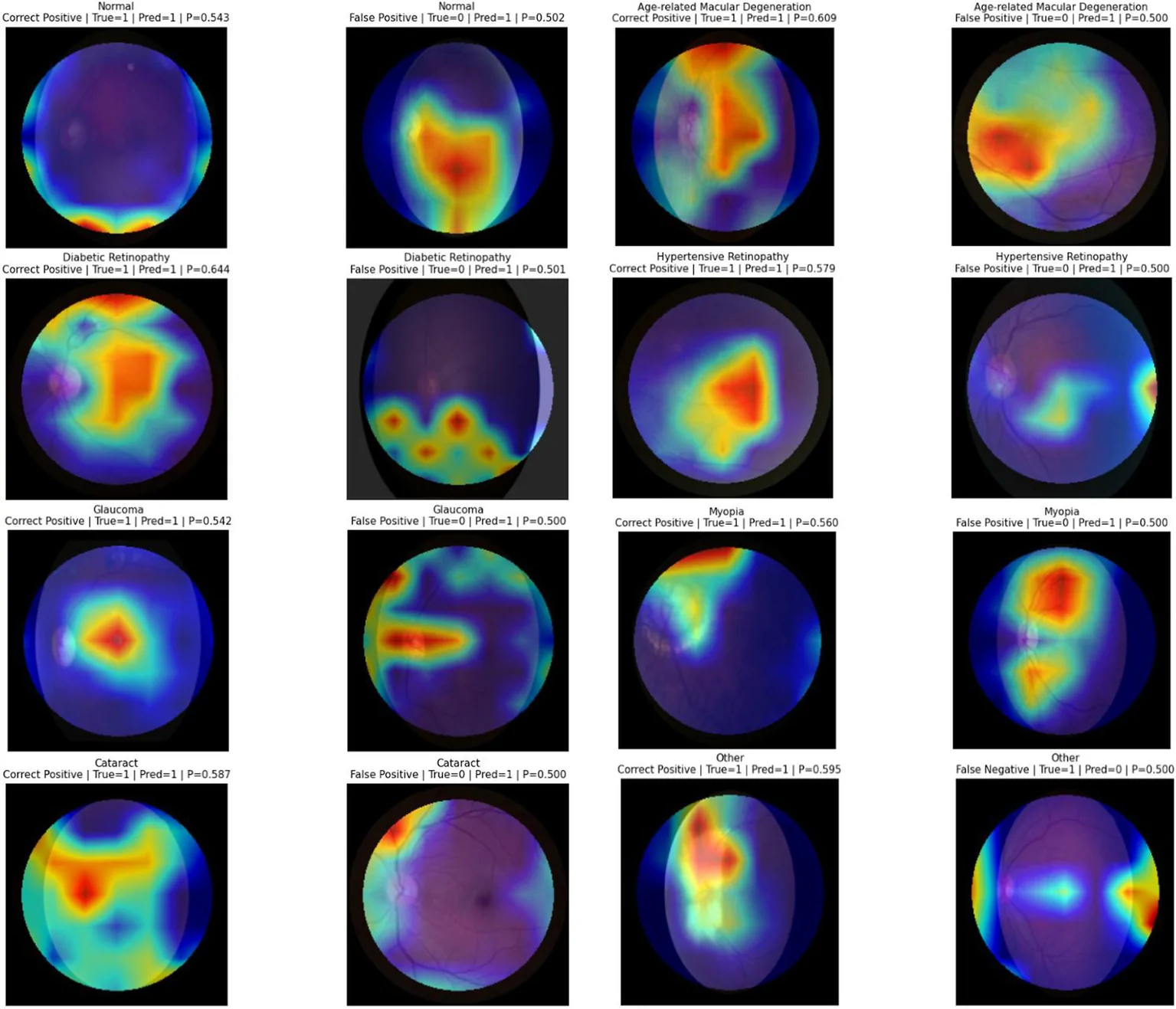
Grad-CAM visualization of correct and incorrect predictions.

When compared to the other existing models, the proposed model obtained a Macro F1-score of 90.36%. Figure 16 describes the comparison of the existing methods with the proposed model.

**Figure 16:**
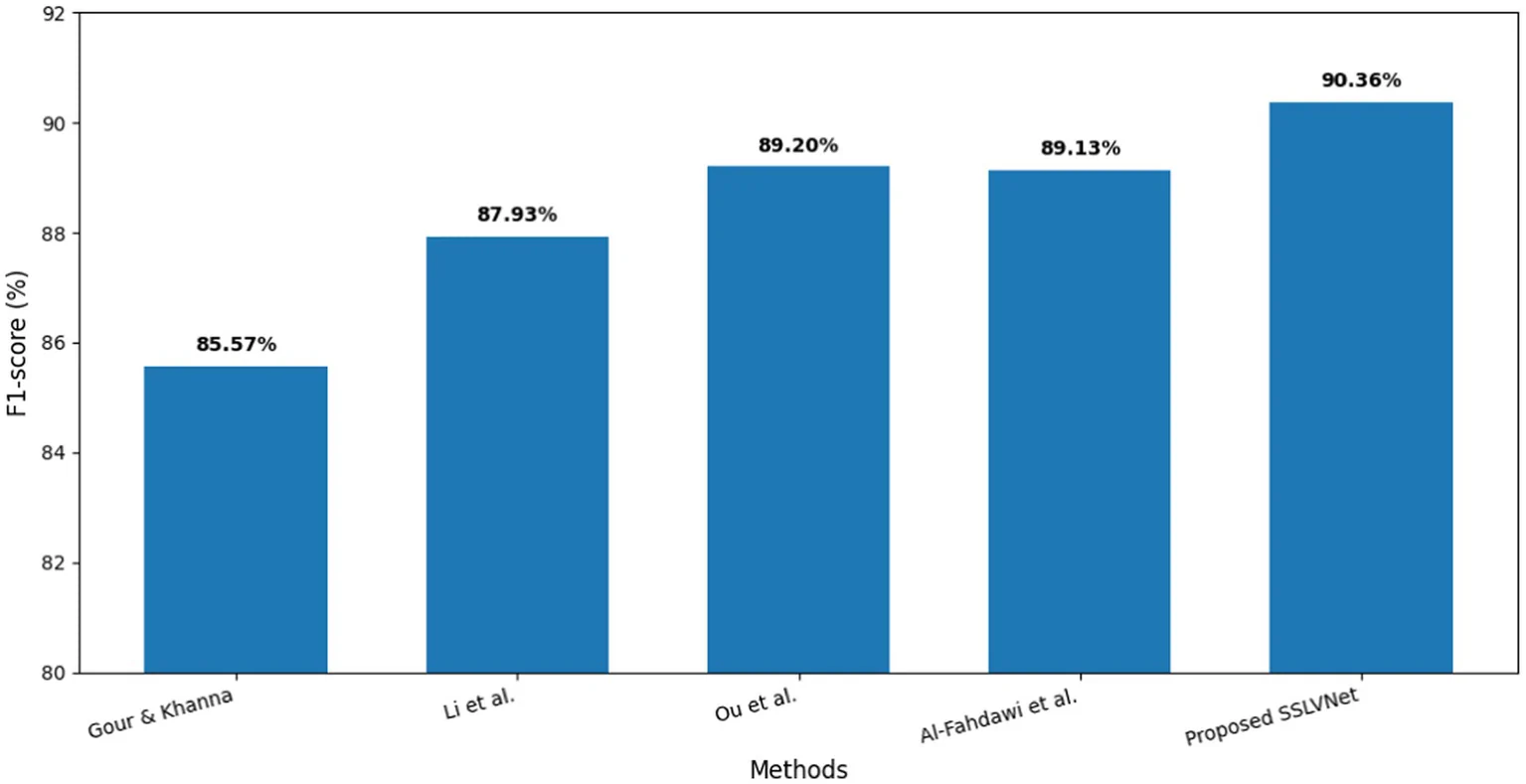
Comparison of existing methods with the proposed SSLVNet.

## 6 Conclusion and future work

A multi-modal automated retinal eye disease classification using the ODIR-5K dataset was implemented. The model combines self-supervised representation learning with hybrid deep neural architectures, including CNNs and ViT, to efficiently capture both localized retinal abnormalities and wider structural designs. Additionally, attention-based mechanisms and graph-oriented feature modeling are incorporated to enhance the understanding of complex lesion relationships within retinal images. Experimental results indicate the proposed system delivers reliable performance across multiple retinal disease categories. Moreover, interpretability is improved through Grad-CAM visualizations that highlight influential retinal regions, while uncertainty estimation increases the confidence and trustworthiness of predictions in clinical use. Further research may also focus on developing lightweight transformer architectures that reduce computational cost while maintaining accuracy. In addition, deploying the model in real-time clinical screening environments and integrating it into hospital decision-support systems would improve practical usability and assist clinicians in the early detection of retinal diseases.

## Data Availability

Publicly available datasets were analyzed in this study. This data can be found here: https://www.kaggle.com/datasets/andrewmvd/ocular-disease-recognition-odir5k.
